# Interaction of Complement Defence Collagens C1q and Mannose-Binding Lectin with BMP-1/Tolloid-like Proteinases

**DOI:** 10.1038/s41598-017-17318-w

**Published:** 2017-12-05

**Authors:** Monique Lacroix, Agnès Tessier, Chantal Dumestre-Pérard, Sandrine Vadon-Le Goff, Evelyne Gout, Leena Bruckner-Tuderman, Dimitra Kiritsi, Alexander Nyström, Sylvie Ricard-Blum, Catherine Moali, David J. S. Hulmes, Nicole M. Thielens

**Affiliations:** 1 Univ. Grenoble Alpes, CNRS, CEA, IBS, F-38000 Grenoble, France; 2 Univ. Lyon, Université Claude Bernard Lyon 1, CNRS, Tissue Biology and Therapeutic Engineering Unit, LBTI, UMR 5305, F-69367 Lyon, France; 3 Laboratoire d’Immunologie, Pôle de Biologie, CHU Grenoble Alpes, 38700 La Tronche, France; 4 BNI group, TIMC-IMAG UMR5525 Université Grenoble Alpes, 38706 La Tronche, France; 5 Department of Dermatology, Medical Center – University of Freiburg, Faculty of Medicine, University of Freiburg, Freiburg, Germany; 6 Univ. Lyon, University Claude Bernard Lyon 1, INSA Lyon, CPE, Institute of Molecular and Supramolecular Chemistry and Biochemistry (ICBMS), UMR 5246, F-69622 Villeurbanne, France

## Abstract

The defence collagens C1q and mannose-binding lectin (MBL) are immune recognition proteins that associate with the serine proteinases C1r/C1s and MBL-associated serine proteases (MASPs) to trigger activation of complement, a major innate immune system. Bone morphogenetic protein-1 (BMP-1)/tolloid-like proteinases (BTPs) are metalloproteinases with major roles in extracellular matrix assembly and growth factor signalling. Despite their different functions, C1r/C1s/MASPs and BTPs share structural similarities, including a specific CUB-EGF-CUB domain arrangement found only in these enzymes that mediates interactions with collagen-like proteins, suggesting a possible functional relationship. Here we investigated the potential interactions between the defence collagens C1q and MBL and the BTPs BMP-1 and mammalian tolloid-like-1 (mTLL-1). C1q and MBL bound to immobilized BMP-1 and mTLL-1 with nanomolar affinities. These interactions involved the collagen-like regions of the defence collagens and were inhibited by pre-incubation of C1q or MBL with their cognate complement proteinases. Soluble BMP-1 and mTLL-1 did not inhibit complement activation and the defence collagens were neither substrates nor inhibitors of BMP-1. Finally, C1q co-localized with BMP-1 in skin biopsies following melanoma excision and from patients with recessive dystrophic epidermolysis bullosa. The observed interactions provide support for a functional link between complement and BTPs during inflammation and tissue repair.

## Introduction

The complement system is a complex extracellular protein cascade that, when triggered by interactions with self or non-self molecules, results in the production of inflammatory mediators and a membrane attack complex that helps destroy invading cells (for recent reviews see^1,2^). It comprises several proteins, including proteases and their substrates/interaction partners, permitting complement activation by one of three pathways (classical, lectin, alternative), all of which converge in the activation of component C3. The classical pathway is triggered by interactions of targets with component C1 which consists of three proteins C1r, C1s and C1q. C1r and C1s are multi-domain serine proteases where two copies of each form a tetramer that associates with C1q, a hexameric recognition protein of the defence collagens family resembling a bouquet of flowers^3^. The lectin pathway is initiated by similar complexes, consisting of other defence collagens, including collectins (mannose-binding lectin (MBL), collectin-10 or collectin-11) and ficolins, bound to MBL-associated serine proteases (MASP-1 and MASP-2)^4^. Binding of the initiating complexes to appropriate targets through the globular recognition domains of the defence collagens triggers sequential activation of the C1r and C1s or MASP-1 and MASP-2 proteases that are bound to the collagen-like stalks of the recognition proteins. A third homologous protease, MASP-3, is found in association with the recognition proteins of the lectin pathway, but it is not involved in triggering activation of the lectin pathway. Activated C1s and MASP-2 are both able to cleave the complement components C4 and C2, which results in the formation of the C3 convertase C4bC2a. The alternative pathway begins with direct activation of component C3 by a C3 convertase assembled from spontaneously hydrolysed C3 and factors B and D on target surfaces^5^. The involvement of MASP-3 in activation of pro-factor D has recently been established^6^.

Bone morphogenetic protein-1 (BMP-1)/tolloid-like proteinases, otherwise known as BTPs, are extracellular zinc-dependent metalloproteinases whose main roles are in extracellular matrix assembly and growth factor signalling^7^. For example, they control collagen assembly by cleavage of propeptides from precursor forms of collagens, lysyl oxidases and small leucine rich proteoglycans, and they activate growth factors by maturation of latent forms or cleavage of growth factor antagonists. They are also involved in angiogenesis and biomineralization^7^. BTP family members in humans include bone morphogenetic protein-1 (BMP-1), mammalian Tolloid (mTLD), and mammalian Tolloid-like-1 (mTLL-1) and -2 (mTLL-2).They have been shown to control many aspects of development, growth and tissue repair, and are involved in diseases such as cancer and fibrosis^7^.

**Figure 1:**
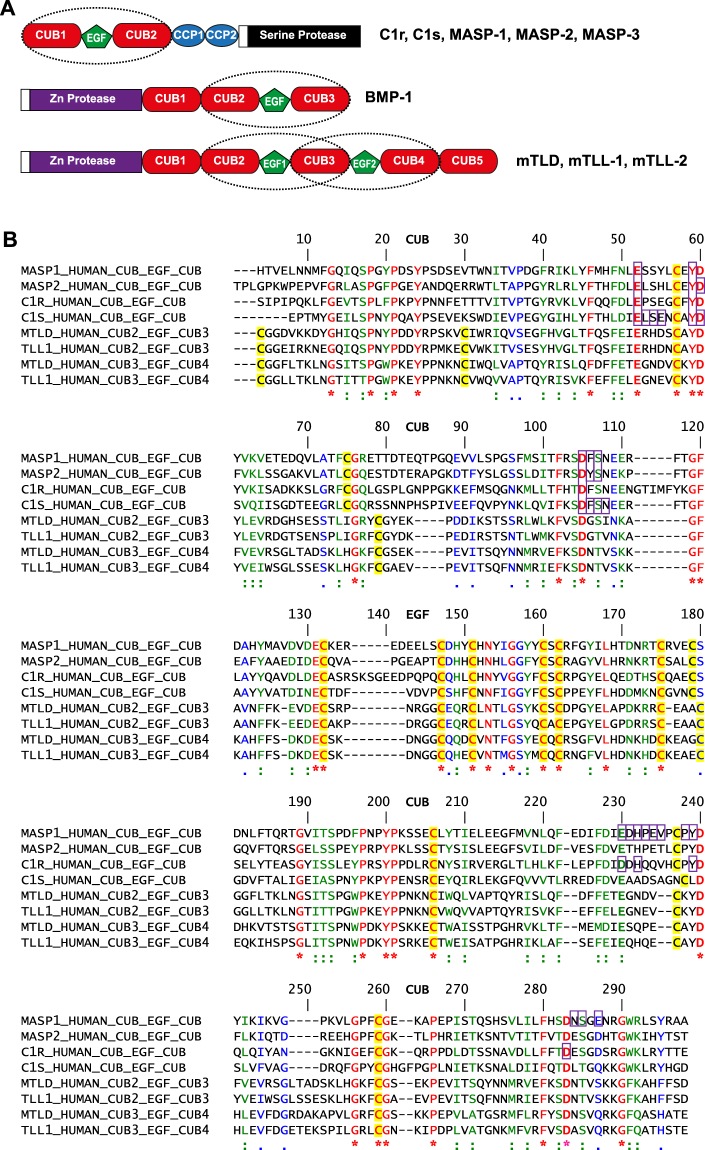
C1r/C1s/MASPs and BTPs are modular proteinases with CUB-EGF-CUB motifs. **(A)** These proteins consist of a protease domain linked to an N-terminal (C1r, C1s, MASPs) or C-terminal (BTPs) non-catalytic region that contains one or two trimodular CUB-EGF-CUB motif(s). CUB: complement C1r/C1s, Uegf, Bmp1; EGF: epidermal growth factor; CCP: complement control protein. Note that BMP-1 and mTLD are products of alternative splicing from the same gene, as is also the case for MASP-1 and MASP-3. The amino acid sequences of BMP-1 and mTLD are identical up to the end of the CUB3 domain, while those of MASP-1 and MASP-3 are identical up to the end of the CCP2 domain. (**B**) Sequence alignment of CUB-EGF-CUB motifs in complement proteases and BTPs. Identical residues are in red (*), strongly similar residues are in green (:) and similar residues are in blue (.). Cysteines are highlighted in yellow. Residues involved in Ca^2+^ binding are in bold. Regions involved in binding to MBL or C1q are boxed^10,15,40,54^.

Despite their different roles, proteins of the complement and BTP systems share many similarities. In particular, though complement proteases are serine proteases and BTPs are metalloproteinases, the non-catalytic domains of C1r, C1s, MASP-1 and MASP-2 and the BTPs include both CUB (complement C1r/C1s, Uegf, Bmp1) and EGF (epidermal growth factor) domains^8,9^ (Fig. 1A). Furthermore, the trimodular CUB-EGF-CUB motif is found in all these proteinases, but nowhere else in mammalian protein databases. This motif plays key roles in the binding of complement proteases to defence collagens, and in the recognition of procollagen substrates by BTPs^7,10,11^. In addition, there is increasing evidence for a link between complement and BTPs during inflammation and tissue repair, as found for example in myocardial infarction^12^ and pulmonary fibrosis^13^. Despite this, to date there have been no studies on possible interactions between BTPs and defence collagens. Here we describe interactions between C1q and MBL and the BTPs BMP-1 and mTLL-1 that provide further support for such a functional connection.

## Results

### The defence collagens C1q and MBL interact with BMP-1 and mTLL-1

**Figure 2:**
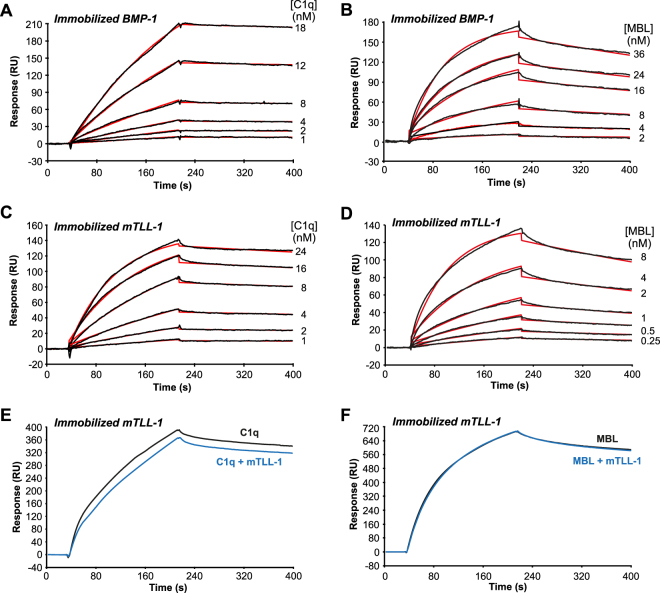
Kinetic analyses of the interaction of C1q or MBL with immobilized BMP-1 or mTLL-1 and competition with soluble mTLL-1 for C1q or MBL binding to immobilized mTLL-1. (**A,B**) Sixty µl of C1q and MBL were injected at the indicated concentrations over immobilized BMP-1 (740 and 850 RU, respectively) in 50 mM triethanolamine-HCl, 145 mM NaCl, 2 mM CaCl_2_, 0.005% surfactant P20, pH 7.4. (**C**,**D**) C1q and MBL were injected over immobilized mTLL-1 (310 and 720 RU) under the same conditions as in (**A**,**B**). Fits shown as red lines were obtained by global fitting of the data using a 1:1 Langmuir binding model. Chi2 values were between 0.6 and 3.6. Each kinetic analysis shown is representative of three (**B**,**C**) and four (**A**,**D**) independent experiments performed on separate sensor chips. (**E**,**F**) C1q and MBL (10 nM) were incubated for 15 min at room temperature in the presence of mTLL-1 (60 nM) before injection over immobilized mTLL-1 (3,400 RU) in 50 mM triethanolamine-HCl, 145 mM NaCl, 2 mM CaCl_2_, 0.005% surfactant P20, pH 7.4. One representative experiment out of two is shown.

The possible interaction of the defence collagens C1q and MBL with BTPs was investigated by surface plasmon resonance spectroscopy (SPR), as previously described to study the interactions between C1q and MBL and their associated complement proteases^10,14,15^. BMP-1 and mTLL-1 were immobilized on the surface of a sensor chip and the defence collagens injected over the immobilized proteinases in the presence of Ca^2+^ ions. Both C1q and MBL bound dose-dependently to immobilized BMP-1 (Fig. 2A,B) and mTLL-1 (Fig. 2C,D). Binding was Ca^2+^-dependent since all interactions were abolished in the presence of EDTA (Supplementary Fig. S1). The kinetic parameters of the interactions were determined by global fitting of the curves using a 1:1 binding model (Fig. 2A–D). BMP-1 interacted with C1q and MBL with high affinity, as reflected by affinity constants (*K*_D_) in the nM range (0.48 and 1.3 nM, respectively), the C1q/BMP-1 complex being slightly more stable than the MBL/BMP-1 complex, as indicated by a 4-fold lower dissociation rate constant (Table 1). The mTLL-1 proteinase also bound to C1q and MBL with kinetic and dissociation constants in the same range as those measured for BMP-1 (Table 1). The *k*_a_ value for the C1q-mTLL1 interaction was slightly lower than that for the C1q-BMP-1 complex and slightly higher in the case of MBL (Table 1). Despite these minor differences, these data indicate a high affinity binding of C1q or MBL to both BMP-1 and mTLL-1.

**Table 1:** Kinetic and equilibrium dissociation constants for binding of C1q and MBL to immobilized BMP-1 and mTLL-1.

Immobilized ligand	Soluble analyte	*k*_a_ (M^−1^ s^−1^)	*k*_d_ (s^−1^)	*K*_D_ (M)	n^a^
BMP-1	C1q	4.4 ± 0.5 × 10^5^	2.1 ± 0.7 × 10^−4^	4.8 ± 1.9 × 10^−10^	5
BMP-1	MBL	7.4 ± 1.4 × 10^5^	8.7 ± 1.7 × 10^−4^	1.3 ± 0.5 × 10^−9^	4
mTLL-1	C1q	3.6 ± 0.9 × 10^5^	3.0 ± 0.9 × 10^−4^	8.9 ± 0.9 × 10^−10^	4
mTLL-1	MBL	1.4 ± 0.2 × 10^6^	8.2 ± 2.6 × 10^−4^	5.8 ± 1.7 × 10^−10^	5
MASP-2	MBL	2.8 ± 0.4 × 10^5^	4.7 ± 1.5 × 10^−4^	1.7 ± 0.3 × 10^−9^	2

^a^Number of separate experiments on different sensor chips.

Values are expressed as mean ± standard error.

Immobilization of C1q and MBL on the surface of a sensor chip and injection of soluble BMP-1 (50 nM) and mTLL-1 (150 nM) did not result in any detectable interaction in the case of C1q and only weak binding in the case of MBL (Supplementary Fig. S2), indicating that the SPR configuration with immobilized BTPs is required for stable interaction with the defence collagens. This is in contrast to published observations on the binding of complement proteases to immobilized defence collagens which showed affinity constants in the nanomolar range: C1s-C1r-C1r-C1s tetramer on C1q (1.5–2.7 nM)^10,11^, MASP-3 dimer on C1q (1.35 nM)^16^, MASP-1, MASP-2 and MASP-3 dimers on MBL (3.2 nM, 2.6 nM and 2.6 nM, respectively)^17^. To check for the effects of immobilization, we also measured binding of C1q and MBL to immobilized MASP-2 (Supplementary Fig. S3). By kinetic analysis (Supplementary Fig. S4), a corresponding affinity constant of 1.7 nM was obtained (Table 1), similar to that found for the reverse configuration.

We next pre-incubated C1q and MBL with soluble BMP-1 or mTLL-1 (molar excess of these proteinases to the defence collagens of 6-fold) before injection over immobilized BMP-1 and mTLL-1. No or very limited inhibition of the interactions was observed, as illustrated in the case of C1q and MBL pre-incubated with soluble mTLL-1 and injected over immobilized mTLL-1 (Fig. 2E,F) or of MBL pre-incubated with soluble BMP-1 and injected over immobilized BMP-1 (Supplementary Fig. S5). These results strongly suggest that stable complexes with BTPs did not form in solution, which is consistent with the observed absence or weakness of interactions between soluble BMP-1 or mTLL-1 and the immobilized defence collagens.

### BMP-1 and mTLL-1 interact with the collagen-like stalks of C1q and with MBL at the complement protease binding site

**Figure 3:**
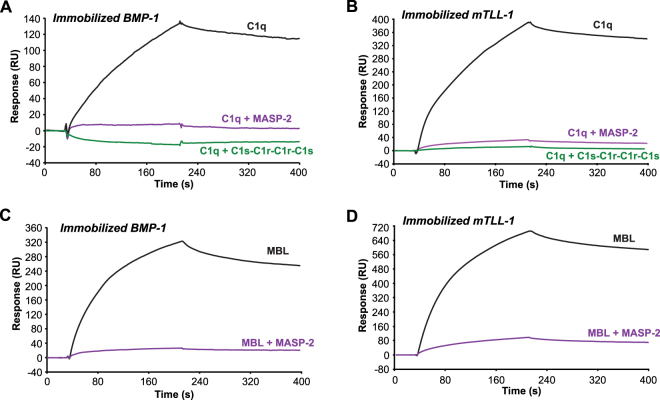
Competition by soluble proteases for binding of C1q and MBL to immobilized BMP-1 or mTLL-1. (**A**,**B**) C1q (10 nM) was incubated 15 min at room temperature in the presence or absence of the C1s-C1r-C1r-C1s tetramer (10 nM) or MASP-2 (60 nM) before injection over immobilized BMP-1 (2,300 RU) and mTLL-1 (3,400 RU) in 50 mM triethanolamine-HCl, 145 mM NaCl, 2 mM CaCl_2_, 0.005% surfactant P20, pH 7.4. (**C**,**D**) MBL (10 nM) was incubated in the presence or absence of MASP-2 (60 nM) before injection over immobilized BMP-1 and mTLL-1 under the same conditions as in (**A**,**B**). (**A**–**D**) One representative experiment out of two is shown.

It has been shown previously that analogous interactions occur between the collagen-like regions of C1q or MBL and the interaction region (CUB1-EGF-CUB2) of C1r and C1s or MASPs, allowing cross-interaction between the subcomponents of the classical and lectin complement pathways^10,11,16^. To investigate whether BMP-1 and mTLL-1 bind to homologous regions within C1q and MBL, we investigated the capacity of the C1s-C1r-C1r-C1s tetramer and the MASP-2 dimer to compete for binding of C1q and MBL to the BTPs. C1q was pre-incubated with C1s-C1r-C1r-C1s or MASP-2 and MBL with MASP-2 and the mixtures were injected over immobilized BMP-1 and mTLL-1. Pre-assembly of the C1 or of the C1q-MASP-2 complex strongly inhibited subsequent interaction with BMP-1 (Fig. 3A) and mTLL-1 (Fig. 3B). Similarly, pre-incubation of MBL with MASP-2 prevented MBL binding to BMP-1 (Fig. 3C) and mTLL-1 (Fig. 3D), suggesting a steric hindrance effect or direct competition of the complement proteases with the BTPs for a common binding site on the collagen stalks of C1q and MBL.

**Figure 4:**
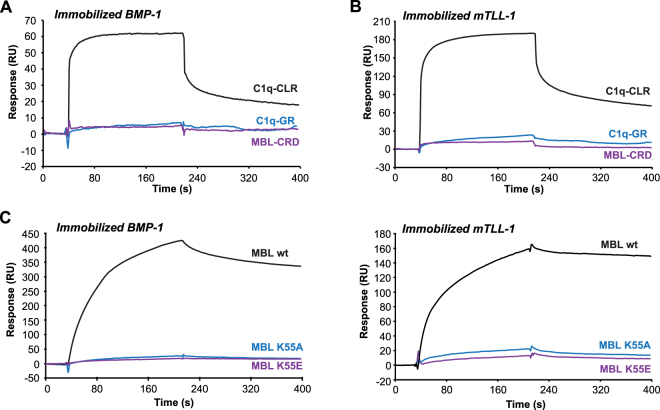
The BMP-1 and mTLL-1 binding site is located in the collagen-like regions of C1q and Lys55 of MBL is essential for the interaction. The collagen-like regions of C1q (CLR), the globular regions of C1q (GR) and the trimeric carbohydrate recognition domain of MBL (CRD) were injected at a concentration of 500 nM over (**A**) immobilized BMP-1 (2,300 RU) and (**B**) mTLL-1 (1,120 RU) in 50 mM triethanolamine-HCl, 145 mM NaCl, 2 mM CaCl_2_, 0.005% surfactant P20, pH 7.4. Wild-type (wt) MBL and its K55A and K55E mutants (16 nM) were injected over (**C**) immobilized BMP-1 (2,300 RU) and (**D**) mTLL-1 (310 RU) under the same conditions as in (**A**). (**A–D**) One representative experiment out of three is shown.

To further locate the BTP binding site on C1q, we used the two functional regions of C1q, *i.e*. its globular regions (GRs) involved in target recognition and its collagen-like regions (CLRs) involved in interaction with C1r and C1s. The CLRs (~190 kDa) comprise six collagen-like triple helices, held together through their N-terminal parts and the GRs (~48 kDa) correspond to the C-terminal trimeric globular regions of the helices. The CLRs bound to immobilized BMP-1 and mTLL-1 whereas no or very weak binding was observed for the GRs (Fig. 4A,B). Similarly, no interaction was detected for the recombinant trimeric carbohydrate recognition domain of MBL (CRD) (Fig. 4A,B).

We have previously generated MBL mutants devoid of MASP-binding ability after mutation of the Lys55 residue of the collagen-like regions^18^. The capacity of the MBL K55A and K55E mutants to interact with immobilized BTPs was compared to that of wild-type MBL. As shown in Fig. 4C,D, both mutations strongly inhibited the interaction, indicating that Lys55 of MBL is essential for the interaction with both BMP-1 and mTLL-1.

### Soluble BMP-1 and mTLL-1 do not inhibit complement activation

**Figure 5:**
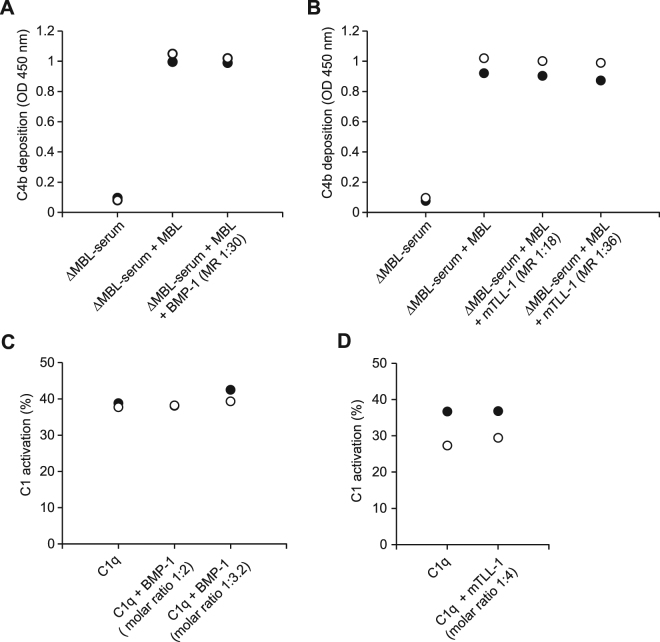
Soluble BMP-1 and mTLL-1 inhibit neither activation of the lectin pathway of human serum complement nor activation of the reconstituted C1 complex. (**A**,**B**) MBL-deficient (Δ-MBL) serum reconstituted with 2 µg/ml MBL, preincubated or not with BMP-1 or mTLL-1 at the indicated molar ratios (MR), was added to mannan-coated microtiter wells. The resulting MASP-2 cleaving activity was measured by a C4b deposition assay. Two independent experiments were performed and each data point is the mean of duplicate (**A**) and quadruplicate measurements (**B**). (**C**,**D**) Complexes (0.25 μM) reconstituted from C1q, pre-incubated or not with BMP-1 or mTLL-1 at the indicated MR, and proenzyme C1s-C1r-C1r-C1s were incubated for 30 min at 30 °C. The extent of C1 self-activation was measured by SDS-PAGE, followed by Western blot analysis using an anti-C1s antibody. The values obtained in two independent experiments are plotted as white and black circles, respectively.

The fact that BMP-1 and mTLL-1 interact with C1q and MBL at the complement protease binding site raised the question of a possible inhibition of complement activation by BTPs. MBL-dependent activation of the lectin complement pathway was measured in mannan-coated microwells by C4b deposition ELISA^19^ using human MBL-deficient serum reconstituted with 2 µg/ml MBL. Pre-incubation of MBL with BMP-1 (Fig. 5A) or mTLL-1 (Fig. 5B) up to a BMP-1/mTLL-1:MBL molar ratio of 30/36:1 yielded no evidence for inhibition of complement activation.

The possible inhibition of the classical complement pathway was also investigated using a C1 activation assay, based on measurement of the self-activation of the C1 complex reconstituted from its isolated purified subcomponents, C1q and the proenzyme C1s-C1r-C1r-C1s tetramer, in the absence of C1-Inhibitor. Incubation of the C1 complex at 30 or 37 °C results in C1r self-activation, followed by C1s activation by activated C1r. Pre-incubation of C1q with BMP-1 (molar BMP-1 excess over C1q up to 3.2-fold) or mTLL-1 (molar excess up to 4-fold) before addition of the proenzyme C1s-C1r-C1r-C1s tetramer and incubation of the resulting complex at 30 °C had no effect on C1 spontaneous activation, determined from the amounts of A and B chains of activated C1s generated (Fig. 5C,D).

These data provided no evidence for an effect of BTPs in solution on complement activation, which is consistent with the observed absence of formation of stable complexes in solution between C1q/MBL and BMP-1/mTLL-1 in the competition experiments.

### Defence collagens are neither substrates nor inhibitors of soluble BMP-1

**Figure 6:**
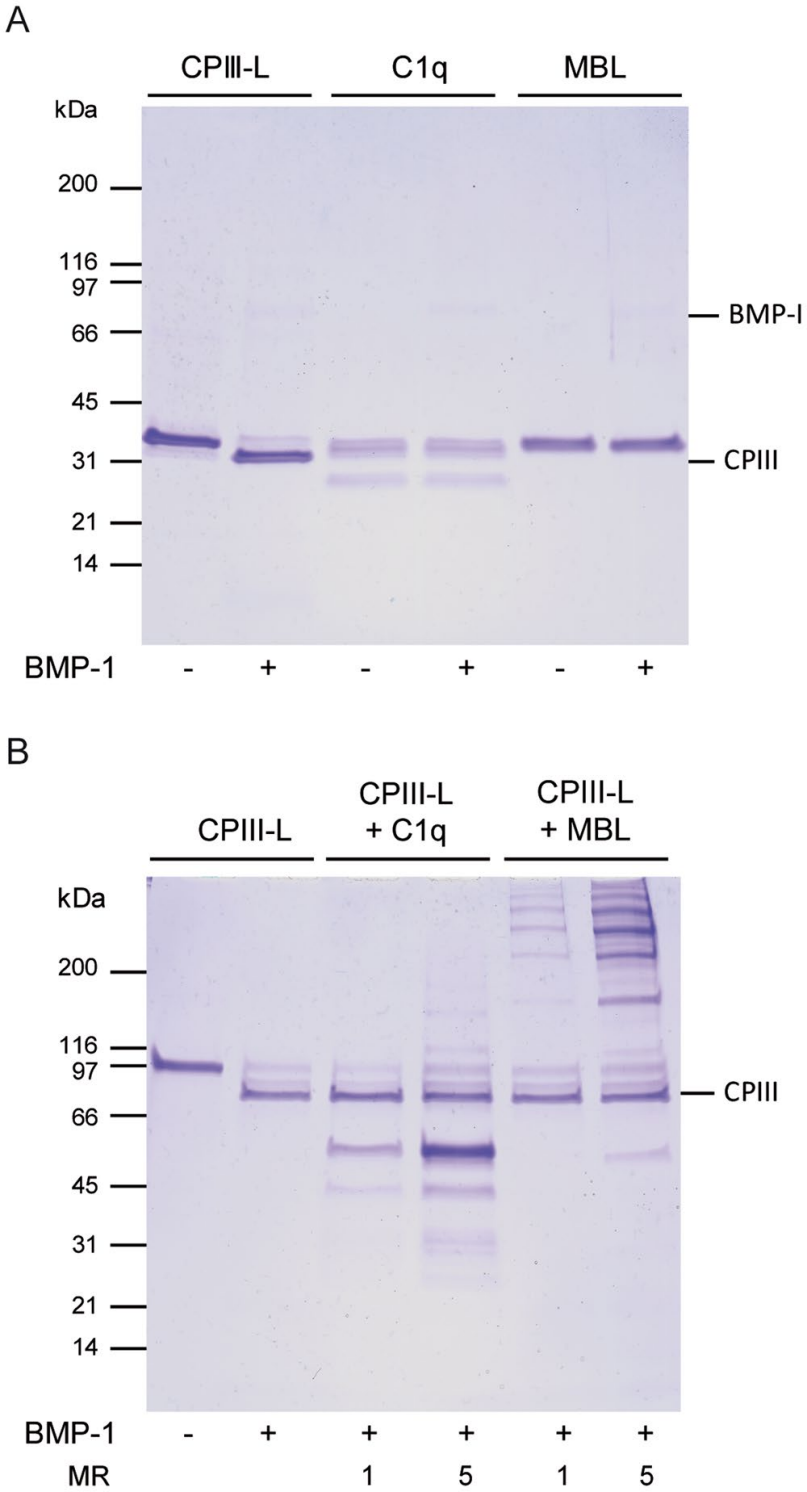
Defence collagens are neither substrates nor inhibitors of BMP-1 in solution. (**A**) CPIII-long (CPIII-L) or defence collagens C1q and MBL (all at 400 nM) were incubated with BMP-1 (20 nM) for 2 h then analyzed by SDS-PAGE (reducing conditions) on a 4–20% acrylamide gradient gel. The band at approximately 30 kDa in lane 2 corresponds to the released C-propeptide (CPIII). Bands for C1q and MBL are unchanged after incubation with BMP-1. (**B**) BMP-1 cleavage of CPIII-long in the absence or presence of defence collagens. Conditions were as in (**A**), except that SDS-PAGE was done in non-reducing conditions. CPIII indicates the released C-propeptide trimer from CPIII-long, the amount of which is unaffected by the presence of C1q or MBL. The molar ratios (MR) of C1q or MBL to CPIII-long are indicated. Whereas C1q analysis under reducing conditions yields a double band containing the A and B chains (31 and 30 kDa, respectively), and the C chain at 26 kDa, analysis under non-reducing conditions yields characteristic A-B and C-C dimers (53 and 48 kDa, respectively). Recombinant MBL migrates as a single band of 30 kDa under reducing conditions and yields a typical ladder-like pattern corresponding to different oligomers under non-reducing conditions. Each gel is representative of three separate experiments. Images are cropped for clarity and conciseness. Full-size gels (including markers) are presented in Supplementary Fig. S6.

To look for possible proteolytic cleavage of the defence collagens by BMP-1, we incubated C1q or MBL with human BMP-1, in conditions known to cleave a standard BMP-1 substrate derived from procollagen III (CPIII-long) which is cleaved between the large C-propeptide trimer (3 x ~30 kDa) and the region (3 x ~3 kDa) comprising the three C-terminal Gly-X-Y triplets of the collagen triple-helix and the C-telopeptide^20^. As shown in Fig. 6A, there was no evidence of BMP-1 cleavage of C1q or MBL in these conditions. We also checked whether pre-incubation with defence collagens might inhibit cleavage of CPIII-long by BMP-1. As shown in Fig. 6B, we found no evidence of inhibition at molar ratios of C1q or MBL to substrate of up to 5:1. These results indicate therefore that the defence collagens are neither substrates nor inhibitors of BMP-1 activity in solution, at least on a standard procollagen III-like substrate.

### BMP-1/C1q co-localization in tissue inflammation

**Figure 7:**
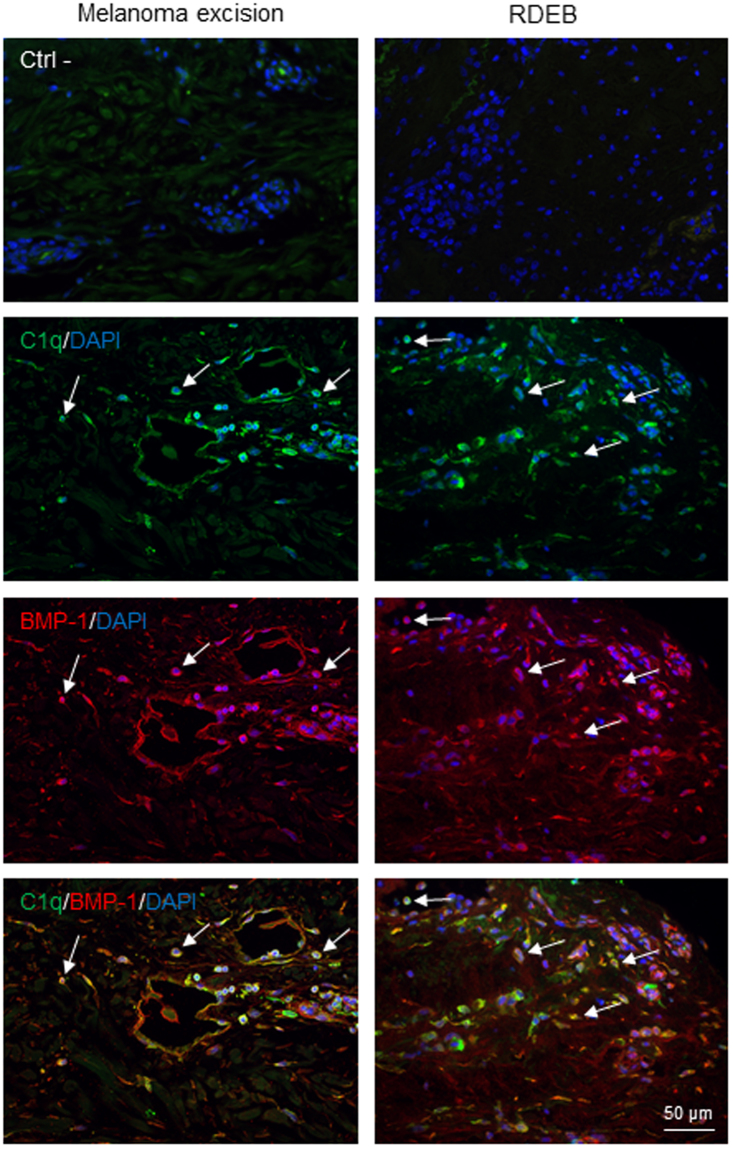
C1q protein colocalizes with BMP-1 in skin biopsies from patients following melanoma excision or with recessive dystrophic epidermolysis bullosa (RDEB). Paraffin-embedded human skin sections were stained with antibodies to C1q (green) and BMP-1 (red). Nuclei were counterstained with DAPI (blue). Images were acquired with a 40X objective (scale bar = 50 µm). Tissue sections were also stained without primary antibody (secondary antibodies only) as negative controls (Ctrl −). Arrows indicate regions positive for C1q and BMP-1 that are co-localized in the lower panels.

To test the possibility of an interaction of C1q with BMP-1 in physiological conditions, we investigated the expression of these two proteins in human skin sections by immunofluorescence. Skin biopsies from clinically non-inflamed regions adjacent to sites of melanoma excision or from patients with recessive dystrophic epidermolysis bullosa (RDEB) were used. RDEB is an inherited skin-blistering disorder caused by mutations in the *COL7A1* gene which encodes type VII collagen, an extracellular matrix (ECM) protein that forms the anchoring fibrils involved in the attachment of the epidermis to the underlying dermis. Skin of RDEB patients exhibits impaired wound healing and scarring and is characterized by strong inflammation^21,22^, as found by the presence of high levels of C1q^23^. As shown in Fig. 7, C1q protein was expressed in biopsies from clinically non-inflamed skin from sites adjacent to melanoma excision and also from RDEB patient skin, as well as BMP-1. Evidence for co-localization of BMP-1 and C1q was found in all cases (Fig. 7). Co-localization of C1q and BMP-1 was further corroborated, using several images (n = 10), by calculation of the Pearson correlation coefficient between C1q and BMP-1 staining. This revealed a moderate to strong co-localization of C1q and BMP-1 staining, as indicated by a correlation coefficient ranging from 0.475 to 0.695 (Supplementary Fig. S7).

## Discussion

In recent years, it has become increasingly clear that the roles of the ECM and innate immunity are closely intertwined^12,24–28^. The defence collagens C1q and MBL play pivotal roles, either as part of the C1 and MBL-MASP complexes where they promote inflammation via the complement cascade, or when, free of such complexes, they contribute locally to the resolution of inflammation prior to tissue repair. C1q and MBL bind to several ECM constituents, including the small leucine-rich repeat proteoglycans (SLRPs) decorin and biglycan^29,30^ and cartilage oligomeric matrix protein (COMP)^31^, with various outcomes in terms of complement activation. Decorin and biglycan bind to both C1q CLRs and GRs and prevent activation of the classical complement pathway; they also bind MBL, but only biglycan inhibits the lectin pathway^30^. Similarly, COMP prevents activation of both pathways by binding to the CLRs of C1q or MBL^31^. In contrast, other SLRPs such as fibromodulin and osteoadherin and the metalloproteinase MT1-MMP have been found to trigger complement activation by binding to the GRs of C1q^29,32^. The ECM proteins fibronectin^33^ and laminin^34^ also bind to C1q, likely through its CLRs, but with unknown effects on complement activation. Finally, MBL and C1q interact with the collagen receptors integrin α2β1^35^ and LAIR-1^36^. The C1q-α2β1 interaction is thought to play a role in mast cell activation and cytokine secretion^35^ whereas C1q binding to LAIR-1 leads to differentiation arrest of dendritic cells (DC) and suppression of DC-derived pro-inflammatory cytokine production^36^.

In addition to interacting with ECM proteins and related receptors, C1q and MBL act as opsonins to facilitate the clearance of apoptotic cells by tissue macrophages and DCs^37,38^ which themselves are major producers of these defence collagens. Macrophages also produce many cytokines, which, depending on macrophage polarization, can be either pro-inflammatory (M1 macrophages) or anti-inflammatory (M2 macrophages). C1q has been shown to regulate the switch to the M2 phenotype^39^, resulting in the production of anti-inflammatory cytokines such as TGF-β which is crucial for ECM homeostasis and tissue repair. As an example of this continuity between innate immunity and ECM, M2 macrophage activation via the IL-4 receptor has recently been shown to enhance tissue repair, including vascularisation and collagen assembly, in a skin wound-healing model^27^. Using a similar model, C1q has also been shown to enhance wound repair by stimulating angiogenesis via interactions with endothelial cell receptors^24^. Finally, the periodontal form of Ehlers-Danlos syndrome (pEDS), characterised by periodontitis and gingival recessions but also skin abnormalities such as easy bruising, fragility and abnormal collagen fibrils, was recently shown to be caused by mutations in the complement proteases C1r and C1s, though the underlying molecular mechanisms are unknown^25^.

In view of these links between innate immunity and the extracellular matrix, as well as the presence of CUB-EGF-CUB motifs in both complement proteases (C1r, C1s, MASPs) and BTPs, we sought to determine whether BMP-1 and mTLL-1 might interact with the complement proteins C1q and MBL. We found that both C1q and MBL were able to bind to immobilized BMP-1 and mTLL-1, with affinities in the nanomolar range. As also found for their cognate binding partners, binding of BMP-1/mTLL-1 to C1q/MBL was Ca^2+^-dependent and to the collagen-like region. Experimental evidence for this was provided by competition experiments using complement proteases, for C1q using separated globular and collagen-like domains and finally for MBL using site-directed Lys55 mutants in the region of the known MASP binding site^18^. Residues analogous to Lys55 have been shown to play similar roles in C1q binding to C1s-C1r-C1r-C1s^16,40^. These observations suggest that BMP-1/mTLL-1 binding to C1q and MBL involve similar interactions to those between these defence collagens and their cognate binding partners, mostly likely involving CUB domains. Comparison of sequences for the trimodular CUB-EGF-CUB motifs found in complement proteases and BTPs (Fig. 1B) shows conservation in BTPs of residues known to be important for complement protease binding to C1q/MBL, particularly the acidic residues that are also involved in Ca^2+^ binding. In contrast, there is less conservation of interacting residues adjacent to these sites.

BTP binding affinities on defence collagens were similar to those previously obtained for complement proteases^10,11,17^, albeit that in the latter case the defence collagens were in the immobilized phase. Control experiments performed here showed that the binding affinities for C1q/MBL on immobilized MASP-2 were similar to those found for the reverse configuration. In contrast, when BMP-1 or mTTL-1 was in the mobile phase, little or no binding was found to immobilized C1q or MBL, despite the fact that mTLL-1, unlike BMP-1, appears to be a dimer in solution^41^. This is reminiscent of previous studies where binding of MBL to the complement receptor CR1, which involves the same protease binding site, was only observed when CR1 was in the immobilized phase^42^. These observations suggest that, unlike for their cognate binding partners, binding of C1q/MBL to BMP-1/mTLL-1 either requires a conformational change induced by immobilization or is a cooperative phenomenon involving multiple BMP-1/mTLL-1 monomers immobilized in close proximity on the sensor chip. Both interpretations are consistent with the observation that soluble BMP-1/mTLL-1 could not compete for C1q/MBL binding to the immobilized BTPs, in contrast to soluble C1s-C1r-C1r-C1s/MASP-2 which strongly inhibited BTP binding to C1q/MBL in either configuration.

Soluble BMP-1 and mTLL-1 did not inhibit activation of the classical and lectin complement pathways and the defence collagens were neither substrates nor inhibitors of BMP-1 in solution, suggesting that the functional consequences of the observed molecular crosstalk are limited to situations where BMP-1 is bound to the matrix. In this regard, the immunofluorescence data showed clear evidence of co-localization of C1q and BMP-1 in biopsies from clinically non-inflamed skin, adjacent to melanoma excision sites and from patients suffering from RDEB. The presence of C1q in the former is expected since C1q has been found to be associated with stromal elements at the invasion edge of several tumours, including melanoma^43^. In an RDEB mouse model, increased expression of C1q has been documented by mass spectrometry and biochemical analysis^23^. The observed co-localization of C1q and BMP-1 suggests that these proteins might interact *in vivo*. Since the *in vitro* data show that binding of defence collagens to BTPs requires the latter to be immobilized on a solid support, this suggests that there may be a similar requirement *in vivo*. As it has been shown that BMP-1 binds to the fibronectin matrix produced by culture human neonatal fibroblasts^44^, one such candidate is fibronectin, which also binds C1q^33^. Thus interactions analogous to the observed *in vitro* interaction between free C1q/MBL and immobilized BTPs might serve to co-localize these proteins *in vivo*, so as to coordinate resolution of inflammation and tissue repair.

## Methods

### Proteins

C1q and the proenzyme form of the C1s-C1r-C1r-C1s tetramer were purified from human serum and quantified as described previously^45,46^. The collagen-like regions (CLRs) and the globular regions (GRs) of C1q were prepared by limited pepsin and collagenase digestion of C1q, respectively, and purified and quantified as described previously^46,47^. Recombinant human mannose-binding lectin (MBL) and its K55A and K55E variants were produced in Freestyle 293-F cells (Thermofisher Scientific) and purified by affinity chromatography on N-acetylglucosamine-agarose (Sigma-Aldrich) as described previously^18^. The molar concentration of MBL was estimated using a molecular weight of 305 kDa, assuming a majority of tetrameric species present in the samples. The recombinant trimeric carbohydrate recognition domain of MBL was produced in a baculovirus-insect cells system and purified as described by Gjelstrup *et al*.^48^. Recombinant human MASP-2 (stabilized in a proenzyme form by mutation of the active site Ser 618 into Ala^17^) was produced in *Drosophila* S2 cells as described for human ficolin-1^49,50^. Briefly, the cDNA encoding MASP-2 was cloned into the pMT/BiP/V5-His A *Drosophila* expression vector (Thermofisher Scientific) and used for stable transfection of S2 *Drosophila* cells. Stable transfectants were expanded in Schneider’s *Drosophila* medium (Thermofisher Scientific) supplemented with 10% (v/v) heat-inactivated foetal calf serum and 500 µg/ml G418. Production of recombinant MASP-2 was induced by adding 750 µM CuSO_4_ to the culture medium without added serum, and the culture supernatants were harvested after 72 h incubation. Recombinant MASP-2 was purified by affinity chromatography on a C1q-Sepharose column, as described for purification of recombinant MASP-3^42^, and its molar concentration estimated using a MW value of 74,160 and an absorption coefficient (A1%, 1 cm) at 280 nm of 15.7.

Recombinant human BMP-1 with a C-terminal FLAG tag and mTLL-1 with a C-terminal 6x-His tag were produced in HEK 293-EBNA cells as described^41,51,52^. The BTP substrate CPIII-long (derived from human procollagen III) was produced in HEK 293 T cells^20^. SDS-PAGE analysis of all purified proteins used is provided in Supplementary Fig. S8.

### Surface plasmon resonance spectroscopy

All experiments were performed on a BIAcore 3000 instrument (GE Healthcare) at 25 °C. Ligands were covalently immobilized on CM5 sensor chips in 10 mM HEPES, 150 mM NaCl, 3 mM EDTA, pH 7.4 containing 0.005% surfactant P20 using amine coupling chemistry according to the manufacturer’s instructions (GE Healthcare). Protein ligands were diluted in 10 mM sodium acetate at the following concentrations and pH values for injection over the activated dextran surface: BMP-1, mTLL-1 and MBL: 5–10, 1.8–7.4 and 25 µg/ml (pH 4.0); C1q: 35 µg/ml (pH 5.0); MASP-2: 17 µg/ml (pH 3.5). Binding was measured at a flow rate of 20 µl/min in 50 mM triethanolamine-HCl, 145 mM NaCl, pH 7.4, containing 0.005% surfactant P20 and 2 mM CaCl_2_ or 3 mM EDTA. Sixty microliters of each soluble analyte at the desired concentrations were injected over the immobilized ligands, and the surfaces were regenerated by 10 µl injections of 10 mM Tris-HCl, 1 M NaCl, 10 mM EDTA, pH 7.4 and, if needed, 10 µl of running buffer containing 3 M MgCl_2_. A control flow cell submitted to all coupling steps without immobilized protein was used as a reference, and the specific binding signal was obtained by subtracting the background signal over the reference surface. For competition assays, C1q and MBL were incubated for 15 min at room temperature with MASP-2, BMP-1 or mTLL-1 before injection over the immobilized proteinases.

Data were analyzed by global fitting to a 1:1 Langmuir binding model of both the association and dissociation phases for at least six analyte concentrations simultaneously, using the BIAevaluation 3.2 software (GE Healthcare). Buffer blanks were subtracted from the datasets used for kinetic analysis (double referencing). The apparent equilibrium dissociation constants (*K*_D_) were calculated from the ratio of the dissociation and association rate constants (*k*_d_/*k*_a_). Chi2 values were below 7 in all cases.

### Complement activation assays

A C4 cleavage ELISA was used to measure human serum complement activation via the MBL-dependent lectin pathway. Activation was measured as described by Dumestre-Pérard *et al*.^19^, using mannan-coated plates and human MBL-deficient serum reconstituted with 2 µg/ml recombinant human MBL (pre-incubated or not with BMP-1 for 30 min at room temperature), except that plates were developed with tetramethylbenzidine (Sigma), the reaction was stopped by 1 N H_2_SO_4_ and absorbance was read at 450 nm.

For the C1 complex activation assay, C1 (0.25 μM) was first reconstituted from purified serum C1q and the proenzyme C1s-C1r-C1r-C1s tetramer^46^. Self-activation was measured by incubating the resulting complexes at 30 °C for 30 min in 50 mM triethanolamine-HCl, 145 mM NaCl, 2.5 mM CaCl_2_, pH 7.4. The extent of C1 activation was determined from the amounts of the A and B chains of activated C1s generated, following Western blot analysis using an anti-C1s antibody and alkaline phosphatase immunodetection^46^. Image acquisition and densitometric analysis were performed using the ChemiDoc MP imager with Image Lab software (Bio-Rad). Pre-incubation of C1q with BMP-1 or mTLL-1 prior to C1 reconstitution was performed at room temperature for 20 min.

### BMP-1 cleavage activity

For BMP-1 cleavage assays, aliquots of purified plasma-derived human C1q (1 mg/ml) and recombinant human MBL (1 mg/ml) were first dialyzed against assay buffer (50 mM Hepes pH 7.4, 150 mM NaCl, 5 mM CaCl_2_). CPIII-long^20^, in the same buffer, was used as a positive control. Reactions (30 µl volume) were set up, in LoBind Eppendorf tubes, using 400 nM substrate (C1q, MBL) and 20 nM recombinant human BMP-1, followed by incubation for 2 h at 37 °C. Reactions were stopped by transfer to ice and addition of 7.5 µl of 5x Laemmli sample buffer (containing dithiothreitol to a final concentration of 50 mM), followed by heating to 100 °C for 3 min. Samples were analyzed by SDS-PAGE using 4–20% gradient gels (BioRad) and staining with Commassie Blue. To look for possible inhibition of BMP-1 cleavage, mini-procollagen III assays were set up as before, but in the absence or presence of C1q or MBL (400 nM or 2 µM for each). Samples for SDS-PAGE were prepared as before, except in non-reducing conditions, and analyzed using 4–20% gradient gels.

### Immunofluorescence analysis of human skin biopsies

Human skin biopsies were obtained using experimental protocols approved by the Ethics Committee of the University of Freiburg (approval number 45/03–110631) in accordance with the declaration of Helsinki and with written informed consent from all participants and/or their legal guardians. Samples were obtained from four patients following melanoma excision (clinically non-inflamed regions adjacent to the sites of excision, to at least ensure safety margins) and from four patients with recessive dystrophic epidermolysis bullosa (RDEB). For immunofluorescence analysis, samples were first fixed in formalin and embedded in paraffin. Sections (4 µm) were then cut, de-paraffinized and re-suspended in xylene and ethanol. Antigens were retrieved by pronase digestion (0.05% solution in PBS, Roche). Sections were blocked in 3% bovine serum albumin (Chem Cruz) diluted in TBS-0.05% Tween 20 and stained with primary and secondary antibodies diluted in blocking solution. Primary antibodies used were mouse monoclonal anti-C1q (Abcam, clone 9A7, ab71089) and rabbit polyclonal anti-BMP-1 directed against active proteinase^53^ (it should be noted that this antibody recognizes both BMP-1 and mTLD as the antigen used is in the catalytic domain which is identical in both proteinases). Secondary antibodies used were, respectively, AlexaFluor 488-conjugated goat anti-mouse IgG and AlexaFluor 594-conjugated goat anti-rabbit IgG (ThermoFisher Scientific) antibodies. Finally, sections were counterstained with 4′,6-diamidino-2-phenylindole (DAPI) and mounted in Fluorescence Mounting Medium (Dako). Stained sections were analyzed with an Axioplan2 fluorescence microscope (Zeiss), capturing images using a black and white camera followed by analysis with ImageJ.

## Electronic supplementary material

Supplementary information
