# Supplementary material for: Interaction of Complement Defence Collagens C1q and Mannose-Binding Lectin with BMP-1/Tolloid-like Proteinases
Source: Sci Rep. 2017 Dec 5;7:16958. doi: 10.1038/s41598-017-17318-w (PMC5717261; doi:10.1038/s41598-017-17318-w)
Supplement: Supplementary file 1 — Supplementary information [file 41598_2017_17318_MOESM1_ESM.pdf]

Supplementary Information

Interaction of Complement Defence Collagens C1q and Mannose-Binding Lectin with BMP-1/Tolloid-like Proteinases

Monique Lacroix, Agnès Tessier, Chantal Dumestre-Pérard, Sandrine Vadon-Le Goff, Evelyne Gout, Leena Bruckner-Tuderman, Dimitra Kiritsi, Alexander Nyström, Sylvie Ricard-Blum, Catherine Moali, David J.S. Hulmes & Nicole M. Thielens

Figures S1-S8

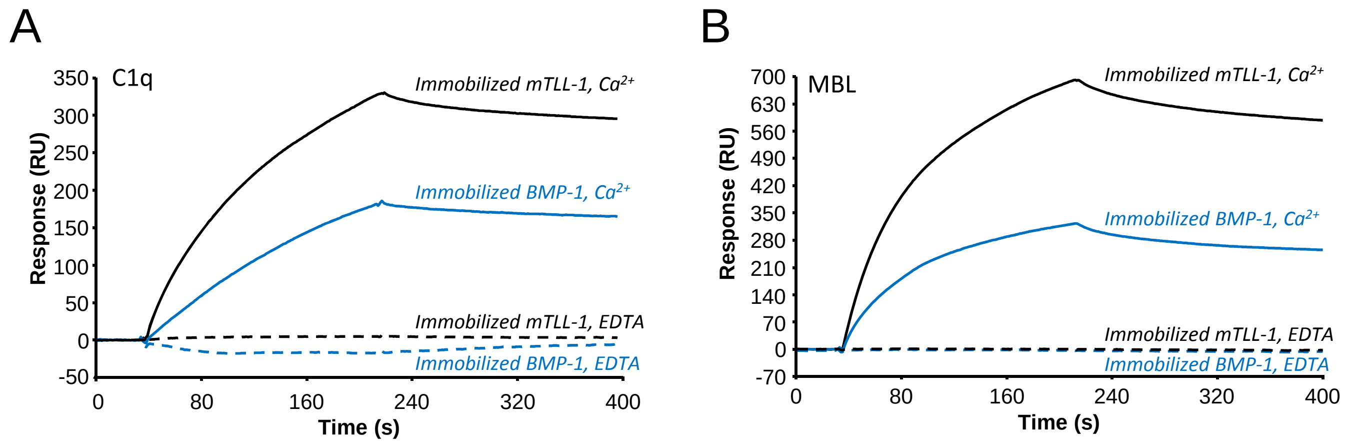

Figure S1. The interaction of C1q and MBL with the immobilized BTPs is calcium-dependent. (**A**) C1q (20 nM) was injected over immobilized BMP-1 (2,300 RU) and mTLL-1 (1,170 RU) in 50 mM triethanolamine-HCl, 145 mM NaCl, 0.005% surfactant P20, pH 7.4 containing 2 mM $CaCl_2$ or 3 mM EDTA. (**B**) MBL (10 nM) was injected over immobilized BMP-1 (2,300 RU) and mTLL-1 (3,400 RU) under the same conditions as in (**A**). (**A,B**) One representative experiment out of three is shown.

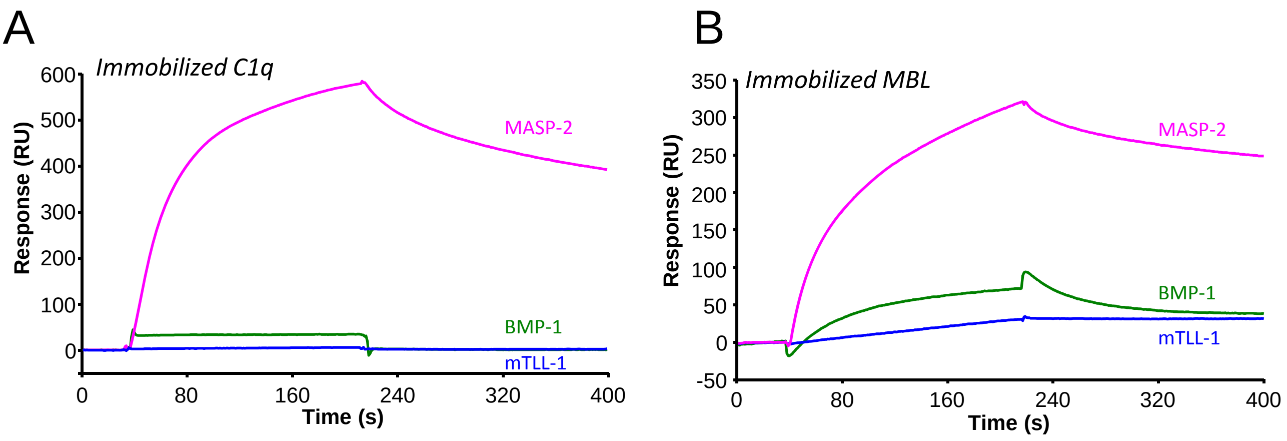

Figure S2. The soluble BTPs interact poorly with immobilized C1q and MBL, contrary to MASP-2. (**A**) BMP-1 (133 nM), mTLL-1 (150 nM) and MASP-2 (47 nM) were injected over immobilized C1q (18,600 RU) in 50 mM triethanolamine-HCl, 145 mM NaCl, 2 mM $CaCl_2$ 0.005% surfactant P20, pH 7.4. (**B**) BMP-1 (50 nM), mTLL-1 (150 nM) and MASP-2 (47 nM) were injected over immobilized MBL (10,600 RU) under the same conditions as in (**A**). (**A,B**) One representative experiment out of two is shown.

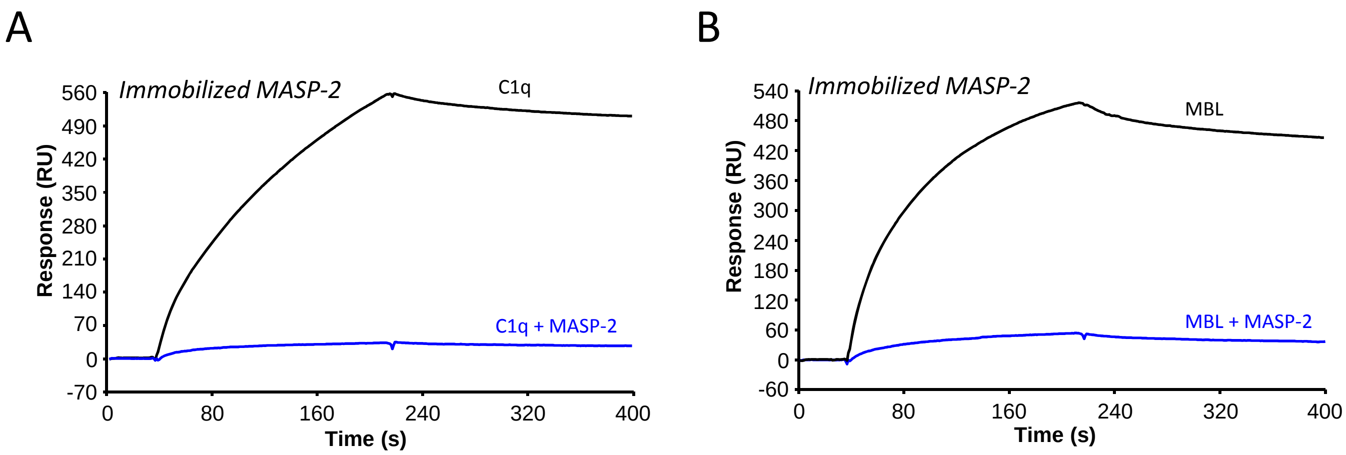

Figure S3. Binding of C1q and MBL to immobilized MASP-2 and competition with soluble MASP-2. (**A**) C1q (20 nM) was incubated 20 min at room temperature with MASP-2 (120 nM) before injection over immobilized MASP-2 (2,900 RU) in 50 mM triethanolamine-HCl, 145 mM NaCl, 2 mM CaCl2, 0.005% surfactant P20, pH 7.4. (**B**) MBL (27 nM) was incubated in the presence of MASP-2 (140 nM) before injection over immobilized MASP-2 under the same conditions as in (**A**). (**A,B**) One representative experiment out of two is shown.

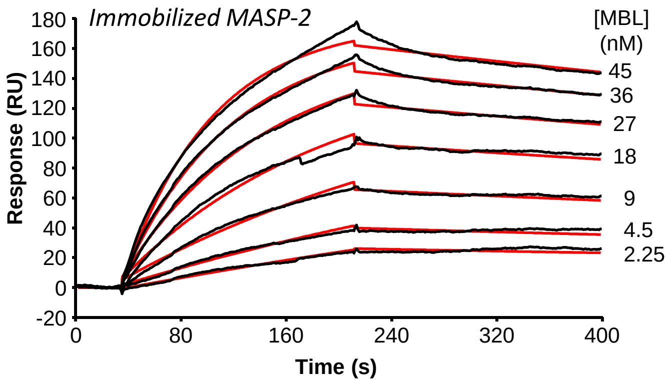

Figure S4. Kinetic analysis of the interaction of MBL with immobilized MASP-2. Sixty µl of MBL were injected at the indicated concentrations over immobilized MASP-2 (3,280 RU) in 50 mM triethanolamine-HCl, 145 mM NaCl, 2 mM $CaCl_2$, 0.005% surfactant P20, pH 7.4. Fits are shown as red lines and were obtained by global fitting of the data using a 1:1 Langmuir binding model (Chi2 = 3.9). The results shown are representative of two independent experiments.

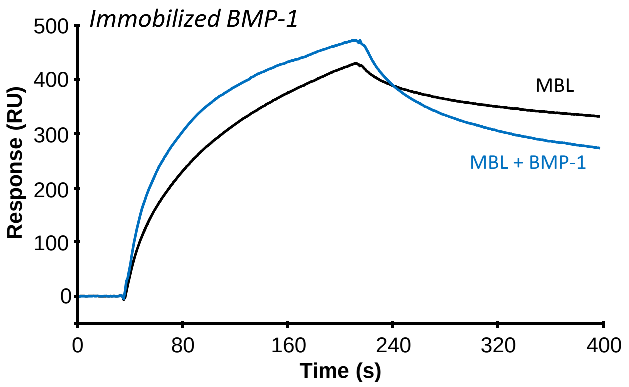

Figure S5. Binding of MBL to immobilized BMP-1 and absence of competition by soluble BMP-1. MBL (20 nM) was incubated 20 min at room temperature with BMP-1 (120 nM) before injection over immobilized BMP-1 (2,300 RU) in 50 mM triethanolamine-HCl, 145 mM NaCl, 2 mM CaCl2, 0.005% surfactant P20, pH 7.4. One representative experiment out of two is shown.

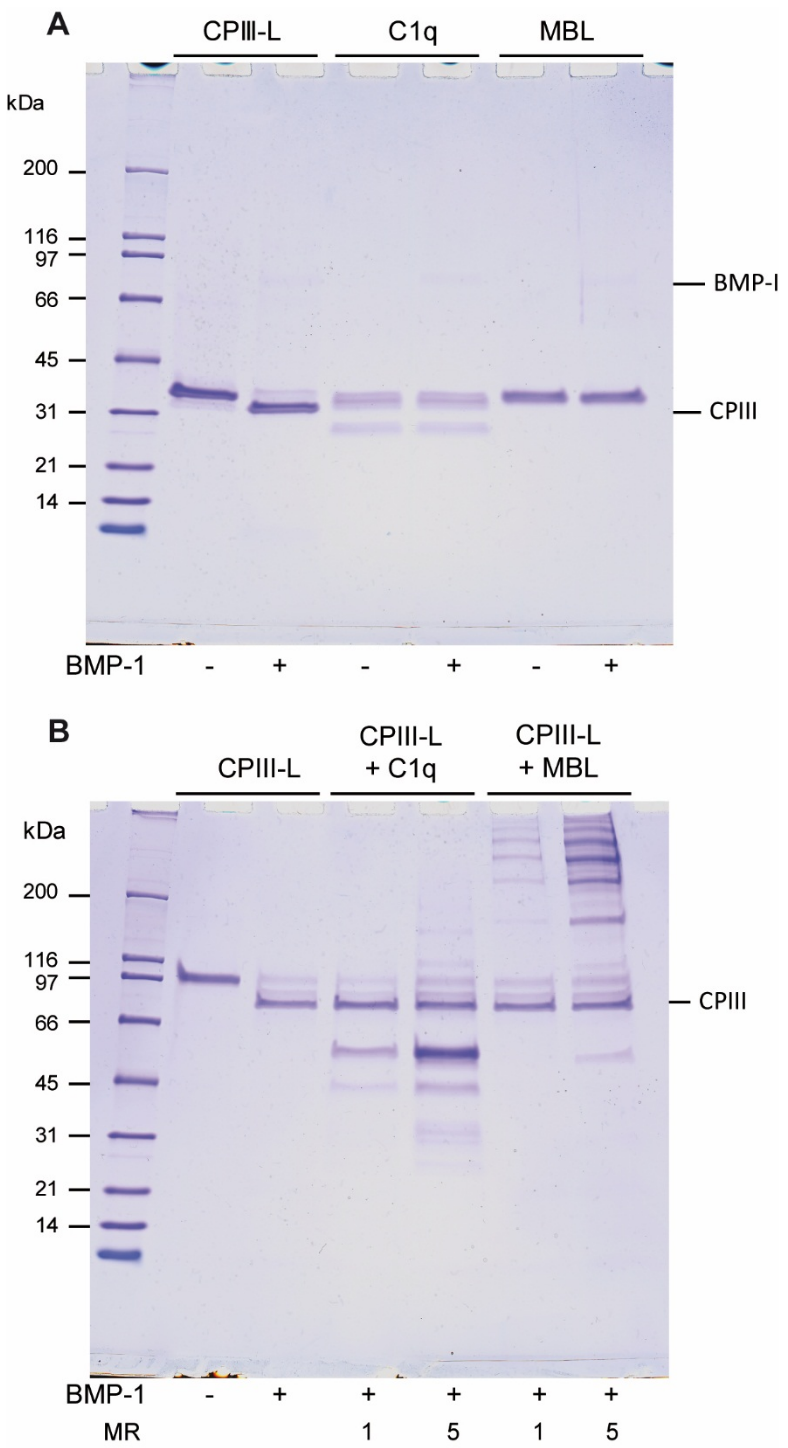

Figure S6. Full-size gels corresponding to the data shown in Fig. 6.

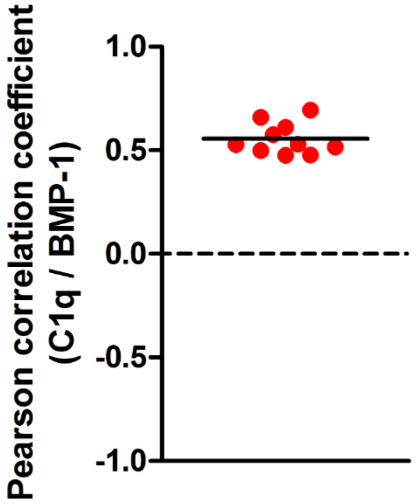

Figure S7. Quantification of C1q/BMP-1 colocalization in inflamed skin by calculation of the Pearson correlation coefficient between C1q and BMP-1 staining (as illustrated in Fig. 7). The Pearson correlation coefficient was calculated using ImageJ and the JACoP plug-in (n=10). The dotted line indicates no correlation between C1q and BMP-1.

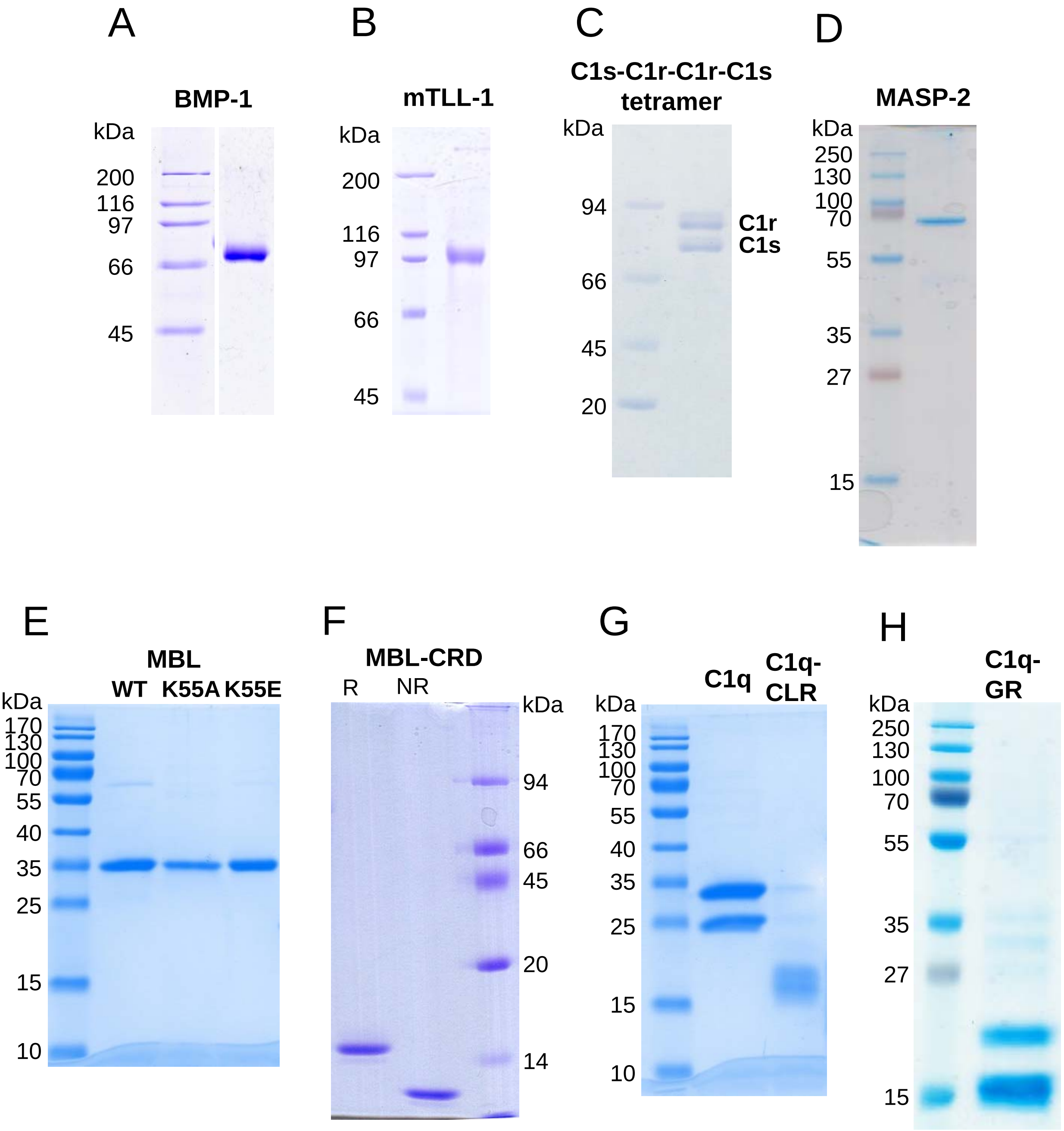

Figure S8. SDS-PAGE analysis and Coomassie Blue staining of the purified proteins used in this article. (**A**) BMP-1 (9% acrylamide gel, non-reducing conditions), (**B**) mTLL-1 (8% acrylamide gel, non-reducing conditions), (**C**) C1s-C1r-C1r-C1s tetramer (10% acrylamide gel, non-reducing conditions), (**D**) MASP-2 (12.5% acrylamide gel, non-reducing conditions), (**E**) MBL, wild-type (WT) and its K55A and K55E mutants (12.5% acrylamide gel, reducing conditions), (**F**) MBL carbohydrate recognition domain (12.5% acrylamide gel, non-reducing (NR) and reducing (R) conditions), (**G**) C1q and its collagen-like region (CLR) (12.5% acrylamide gel, reducing conditions), (**H**) C1q globular region (GR) (12.5% acrylamide gel, reducing conditions).

.
